# A smart magnetic hydrogel containing exosome promotes osteogenic commitment of human adipose-derived mesenchymal stem cells

**DOI:** 10.22038/IJBMS.2022.64682.14237

**Published:** 2022-09

**Authors:** Fatemeh Sadeghian-Nodoushan, Habib Nikukar, Masoud Soleimani, Azarmidokht Jalali- Jahromi, Simzar Hosseinzadeh, Arash Khojasteh

**Affiliations:** 1 Department of Tissue Engineering and Applied Cell Sciences, School of Advanced Technologies in Medicine, Shahid Beheshti University of Medical Sciences, Tehran, Iran; 2 Research and Clinical Center for Infertility, Yazd Reproductive Sciences Institute, Shahid Sadoughi University of Medical Sciences, Yazd, Iran; 3 Medical Nanotechnology and Tissue Engineering Research Center, Yazd Reproductive Sciences Institute, Shahid Sadoughi University of Medical Sciences, Yazd, Iran; 4 Medical Nanotechnology and Tissue Engineering Research Center, Shahid Beheshti University of Medical Sciences, Tehran, Iran; 5 Department of Health and Medical Sciences, University of Antwerp, Antwerp, Belgium

**Keywords:** Exosomes, Magnetic vanoparticles, Mesenchymal stem cells, Scaffold, Tissue engineering

## Abstract

**Objective(s):**

Exosomes, as nano-sized extracellular vehicles acting as cell-to-cell communicators, are novel promising therapeutics in the area of bone tissue engineering. Moreover, magnetic nanoparticles, whose integration with other appropriate components is viewed as an intriguing approach to strengthen bone tissue engineering efficacy. We investigated the effect of magnetic enriched with exosomes on osteogenic differentiation.

**Materials and Methods:**

Exosomes were isolated from human adipose-derived mesenchymal stem cells by Exo-spin™ kit (MSC-EX). Alginate (Alg) scaffold containing 1% (w/w) cobalt ferrite nanoparticles (CoFe2O4) was produced. MSC-EX were gently loaded onto Alg and Alg-cobalt ferrite (Alg-CF) scaffolds yielding Alg-EX and Alg-CF-EX scaffolds. The effects of MSC-Ex and magnetic hydrogel composite under an external static magnetic field (SMF) on proliferation and differentiation of MSCs were evaluated by alkaline phosphatase (ALP) activity measurement, alizarin red staining, and energy dispersive X-ray (EDX) analysis.

**Results:**

Our results showed that Alg and Alg-CF scaffolds were not only cytotoxic but also supported AdMSCs proliferation. MSC-EX loading of the scaffolds enhanced AdMSCs proliferation significantly. According to the results, Alg-CF-EX scaffolds under magnetic stimulation exhibited the most potent effect on osteogenic differentiation of cultured AdMSCs as evidenced by higher ALP activity and mineralization.

**Conclusion:**

We provided evidence that the combination of Alg hydrogel, CFNPs, and MSC-EX resulted in the construction of a bone tissue-engineering scaffold that highly supports the osteogenic commitment of MSCs.

## Introduction

Mesenchymal stem cells (MSCs) have been extensively investigated for tissue repair and regeneration (1, 2). Direct use of these cells has some remarkable limitations such as phenotypic alterations in MSCs during cellular expansion, time-consuming process of cell culture, low homing efficiency of the injected MSCs as well as low survival rate of locally transplanted MSCs (3). As demonstrated by recent evidence, positive therapeutic effects of MSCs are suggested to be largely facilitated by paracrine mechanisms to promote activities of tissue-resident recipient cells (4, 5). Exosomes are naturally secreted nano-vesicles ranging from 40–150 nm in size. These nano-carriers that originate from multivesicular bodies play crucial roles in intercellular communication through transferring proteins and genetic information to target cells (6). MSC-derived exosomes (MSC-EX) have shown notable therapeutic effects in various disease models in the absence of their cellular sources, e.g., MSCs. These nanoparticles have been shown to exert similar functional impacts to the MSCs from which they originate without considerable adverse effects (7, 8). The combination of exosomes with hydrogels can provide several benefits such as preventing exosome washout and maintaining their local concentration. In addition, exosome-loaded hydrogels can be directly employed at or near the treatment site, features that provide a more targeted delivery with reduced exosome dosage (9). In this regard, hydrogels containing high water content closely mimic the extracellular matrix microenvironment. Due to their characteristic traits such as low toxicity, gelling capacity, low cost, and high availability, Alginate hydrogels can be viewed as some of the most popular biopolymers for bone tissue engineering (10). However, alginate has some weak points hindering its extensive use for scaffold preparation including uncontrolled biodegradation, weak mechanical properties, and inadequate interactions with cells (11). 

Recently, magnetic nanoparticles (MNPs) have attracted rising interest in the field of biomedicine. For instance, MNPs have been widely applied as drug carriers to facilitate targeted drug delivery (12) as contrast agents to improve magnetic resonance imaging (MRI) for medical diagnosis (13) and as thermogenic agents for hyperthermia tumor therapy (14). Also, these nanoparticles can be contemplated as potential tools for bone tissue engineering. MNPs, either used in combination with an external magnetic field or alone, can boost bone regeneration. Magnetic materials can be incorporated into scaffolds to promote cell growth and bone formation (15). One of them, cobalt ferrite (CoFe2O4), as spinel ferrites, which can create improved magnetic properties, are also of high interest (16). 

In the present study, we aimed to develop a cell-free bone engineering scaffold comprising alginate hydrogel, cobalt ferrite nanoparticles (CFNPs), and exosomes isolated from adipose-derived MSCs (AdMSC-EX) and investigate its osteoinductive potential *in vitro*. Indeed, we prepared a novel bone regeneration scaffold combining a hydrogel scaffold with MNPs and MSC-EX, as three different elements stimulating bone tissue regeneration. Furthermore, we will investigate the synergistic effect between the magnetic scaffold and exosome osteogenic differentiation. 

## Materials and Methods

***Isolation of adipose-derived mesenchymal stem cells (AdMSCs)***

In this experimental study, to isolate adipose-derived MSCs (AdMSCs), adipose tissue samples were collected under sterile conditions from patients 16– 45 years old, undergoing general abdominal operation in Shahid Sadoughi Hospital, Yazd, Iran, after obtaining informed consent. All procedures were approved by Shahid Beheshti University of Medical Sciences Ethical Committee, Tehran, Iran (Ethic code “IR.SBMU.REC.1399.020”). The samples in sterile phosphate-buffered saline (PBS) were transferred to the research laboratory of Yazd Reproductive Sciences Institute, Shahid Sadoughi University of Medical Sciences. Tissue samples were first ground with a sterile surgical blade. Then, they were treated with collagenase I (1.5 mg/ml, Gibco-Invitrogen, Grand Island, NY, USA) and incubated for 20 min at 37 °C to yield a homogenous suspension. Afterward, the resultant suspensions were centrifuged at 2000 RPM for 20 min. Cell pellets were cultured in Dulbecco’s Modified Eagle Medium high glucose (DMEM-HG, Gibco-Invitrogen) supplemented with 20% fetal bovine serum (FBS, Gibco-Invitrogen), 100 µg/ml streptomycin and 100 IU/ml penicillin in a standard incubator with 37 °C humidified air and 5% CO_2_. culture media were changed every 3–4 days until the cells grew about 90% in confluency. The cells were passaged at the confluence of 90% using 0.25% trypsin-EDTA (Ethylenediaminetetraacetic acid). Characterization of the obtained AdMSCs was performed by a combination of morphologic, flow cytometric, and differentiation analysis (adipogenic and osteogenic). For flow cytometric characterization, AdMSCs were evaluated for cell surface expression of different markers including cluster of differentiation (CD34, CD44, CD45, CD73, CD90, and CD105, Abcam). The cells at passage 3 (P3) were analyzed for their ability to differentiate into osteoblasts and adipocytes. For this, the cells were cultured with the induction medium in 6-well plates. For osteogenic differentiation, stem cells were first seeded in each well of a 6-well plate at a density of 50000 cells/well. When the cells were 90% in confluency, the culture medium was replaced with osteogenic induction medium (DMEM supplemented with 10% FBS+ 7^-10^ M dexamethasone+ 10 mM β-glycerophosphate and 50 µg/ml ascorbic acid-2-phosphate (Sigma). After 21 days, mineral deposition was assessed by Alizarin red staining (Sigma). For adipogenic differentiation, cells were cultured with the adipogenic medium (containing 50 µg/ml ascorbic-2-phosphate+ 7^-10^ M dexamethasone and 50 µg/ml indomethacin (Sigma) for 21 days. Finally, the cells were stained by Oil Red O (Sigma) to assess adipogenesis.

***Exosome isolation and characterization***

MSCs were cultured in T-75 flasks. When the cells grew about 80% in confluency, culture media were replaced with DMEM media supplemented with 10% exosome-free FBS. The conditioned media were collected after 48 hr and stored at -20 °C. Before exosome isolation, the collected conditioned media were concentrated using Amicon Ultra centrifugal filters. Exosomes were isolated using an Exo-spin kit (Cell Guidance Systems, Cambridge, UK) in accordance with the manufacturer’s instructions. Transmission electron microscopy (TEM) analysis was carried out to observe the morphology of the isolated exosomes. Exosome suspension was first fixed using 1% glutaraldehyde and a drop was placed on a carbon-coated grid and left at RT to dry. The grids were then washed with sterile PBS twice, followed by staining with 1% uranyl acetate for 10 min. The samples were analyzed using an Em208s 100kv (Philips, Netherlands). In the next step of exosome characterization, dynamic light scattering (DLS) was used for determining the size distribution of the isolated exosomes by means of a Malvern Zeta sizes Nano ZS (Malvern Instruments, UK). Isolated exosomes were quantified based on their total protein concentration by Bradford assay protocol using bovine serum albumin (BSA) as standard (17). 

***Scaffold construction***

**Figure 1:**
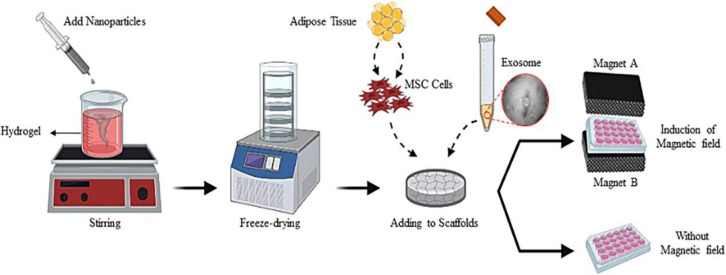
Experimental design to analyze cobalt ferrite nanoparticles (CFNPs) and mesenchymal stem cell-derived exosomes (MSC-EX) and explore their *in vitro *osteoinductive potential

To synthesize CFNPs, aqueous solution (concentration) of ferric nitrate (Fe (NO_3_)_3_) was drop-wisely added to cobalt nitrate (Co (NO_3_)_2_) aqueous solution. The pH of the resultant solution was adjusted to 11 by addition of ammonia solution. Then*, *polyvinyl pyrrolidone (PVP) solution was added followed by stirring for 15 min. Finally, the obtained solution was placed in a 180 °C oven for 3 hr. To construct alginate (Alg) scaffolds, a 1% aqueous solution of sodium alginate (Sigma) was first prepared and stirred. Then, this solution was gently added to calcium chloride solution (3%) and the mixture was left at RT for 15 min until gel formation. The remaining calcium chloride solution was removed and the prepared hydrogels (Alg scaffold) were freeze-dried. For construction of Alg-CF scaffolds, different concentrations of CFNPs were added prior to freeze-drying (Figure 1). 

***Scaffold characterization***

*Scanning electron microscopy (SEM), TEM, and DLS*

To observe the cross-sectional morphology of the prepared scaffolds, SEM analysis was conducted (ZEISS DSM 960A Oberkochen, Germany). The samples were coated with gold and the analysis was accomplished at an accelerating voltage of 30 kV. Also, the synthesized nanoparticles were analyzed for their morphology and size distribution by TEM and DLS, respectively.

*Fourier transform infrared spectroscopy (FTIR)*

To evaluate the chemical integrity of the prepared scaffolds, freeze-dried scaffolds were first ground and the powder was used for FITR analysis. FTIR estimations were recorded within the wavelength range of 400–4000 cm^-1^ using an FTIR spectrometer (Equinox 55, Bruker, Germany).

*X-ray diffraction (XRD) analysis*

XRD is a useful technique to confirm the crystalline or amorphous nature of materials. XRD patterns were registered (Rigaku D/MAX 250, Tokyo, Japan) in the 2θ range of 5–50°.

*Vibrating sample magnetometer (VSM) analysis*

Magnetic evaluation of Alg-CF scaffolds with different concentrations of CFNPs (0.001, 0.01, and 0.1 %v/w) was carried out on a magnetometer at the physiological temperature (310 K) at Kashan University, Iran. 

***Porosity measurement***

Dry weights of prepared Alg and Alg-CF scaffolds were designated as W_S. _A 10 ml centrifuge tube was filled with ethanol and weighed as W_1_. Specimens of each scaffold were immersed in the ethanol-containing tubes and the total weights were recorded as W_2_. Then the scaffolds were taken out and the remaining mass of ethanol and 10 ml tubes after scaffold removal were designated as W_3_. Finally, porosity measurement (%) was performed using the following equation: Porosity (%) = (W_2_ – W_3_ – W_S_) ∕ (W_1_ – W_3_) × 100

***Contact angle measurement***

The hydrophilicity of the scaffold network is evaluated by measuring the angle (θ) between the surface of the scaffold with the line tangent to the water droplet. To do this, one droplet of distilled water (5 µl) was placed on the surface of specimens of each scaffold and after 3 sec, photographs were taken. In order to measure contact angles, the Digimizer software (Version 5.4.6) was utilized. 

***Compressive strength test***

The mechanical fate of the scaffolds was analyzed by the compressive strength test. The compressive strength of the scaffolds was measured according to the ASTM F2150-7 standard by means of a Zwick/Roel machine (HCT 25/400, 25 KN load cell, Zwick/Roell Co., Germany). The elastic modulus size of the scaffold was calculated from the elastic area line slope of the obtained curve. Scaffold specimens were loaded at the speed of 1 mm/min.

***AdMSCs monitoring in the structure of scaffolds***

Alg and Alg-CF scaffolds were washed with 70% alcohol three times and then exposed to UV light for one hour. Then, AdMSCs (50,000 cells) were gently seeded on the surfaces of Alg and Alg-CF scaffolds and incubated at 37 °C in DMEM supplemented with 10% FBS. After culture, scaffolds were fixed using glutaraldehyde solution (2.5% in PBS) for 2 hr followed by dehydration in serial alcohol concentration. The scaffolds were characterized by SEM. Also, 4′, 6-diamidino-2-phenylindole (DAPI) staining was carried out on days 7 and 14 to confirm the adhesion and presence of cells on the scaffolds. DAPI solution was added to the cultures and after that, the scaffolds were observed by a fluorescent microscope. 

***Cell viability assay***

To evaluate the cytocompatibility of the scaffolds, an MTT assay was performed(18). To this end, Alg and Alg-CF scaffolds with different concentrations of CFNPs (0.01, 0.03, 0.05, 0.07, and 0.1% w/v) were prepared. To investigate the effect of a magnetic field on AdMSC proliferation, some of the wells were stimulated by a static magnetic field (SMF) with a magnetic field strength of 125 mT. After scaffold sterilization, in every well, a total number of 50,000 AdMSCs were gently seeded on the scaffolds. Cell viability was assessed after 4, 7, and 14 days of incubation in a standard cell culture incubator. To examine the effects of MSC-EX on the viability and proliferation of the cells, 100 µl volumes of the isolated MSC-EX were loaded onto Alg and Alg-CF scaffolds to yield Alg-EX and Alg-CF-EX scaffolds. Similar to the previous section, an MTT assay was conducted., these experiments were performed under both non-induced and induced conditions. In wells considered as the negative control, only AdMSCs were cultured. All experiments were repeated at least in triplicates.

***Alkaline phosphatase activity***

Alkaline phosphatase (ALP) is involved in the catalysis of p-nitrophenolphosphate hydrolysis into p-nitrophenol and phosphate. ALP activity in different groups was estimated on days 7, 14, and 21 after addition of osteogenic media. To measure ALP activity at every time interval, the adherent cells were lysed for 15 min with CelLytic^TM^ (Sigma-Aldrich). After centrifugation at 20,000 g for 10 min, supernatants were transferred to another tube. Then, Alkaline Phosphatase Yellow (pNPP) Liquid Substrate (Sigma-Aldrich), as ALP substrate solution, was added at a 1:1 ratio and the tube was incubated in the dark at room temperature for 30 min. Afterward, NaOH solution (3 M) at an ALP substrate was added and optical density (OD) was read at 405 nm.

***Extracellular matrix mineralization***

Alizarin red is an organic compound capable of specifically staining the mineralized matrix of the cultured cells. The intensity of red staining corresponds to the level of cell-matrix mineralization and osteoblast formation. The test was performed on day 21 after osteoinduction. After removal of osteogenic media and washing with PBS, the cells were fixed with 4% paraformaldehyde at room temperature for 1 hr. Subsequently, 1 ml of alizarin red solution (40 mM, pH 4.1) was added to each well. After 1 hr, the wells were rinsed with PBS. Finally, after the addition of acetic acid, the ODs of the wells were read at 405 nm.

***Dispersive x-ray (EDX) analysis***

SEM-EDX analysis was performed on day 21 to detect and confirm mineralization (calcium and phosphorus content) following osteogenic differentiation. Cultures were first fixed in 4% paraformaldehyde at room temperature. SEM-related images were taken using an energy-dispersive X-ray spectrometer (Thermo Electron, Noran System) which was attached to the JEOL JSM-6500F microscope. Surfaces were analyzed at 15kV for 5 min.

***Statistical analysis***

The acquired data were expressed as Mean ± standard deviation (SD). The data of each test were obtained from three independent experiments. Analysis of the data was performed using GraphPad Prism software (Version 8.0.1). To make comparisons between the groups, one-way ANOVA followed by Tukey’s *post hoc* test was used. For all tests, statistically significant differences were accepted for *P*-values less than 0.05

## Results

***Characterization of isolated AdMSCs***

**Figure 2:**
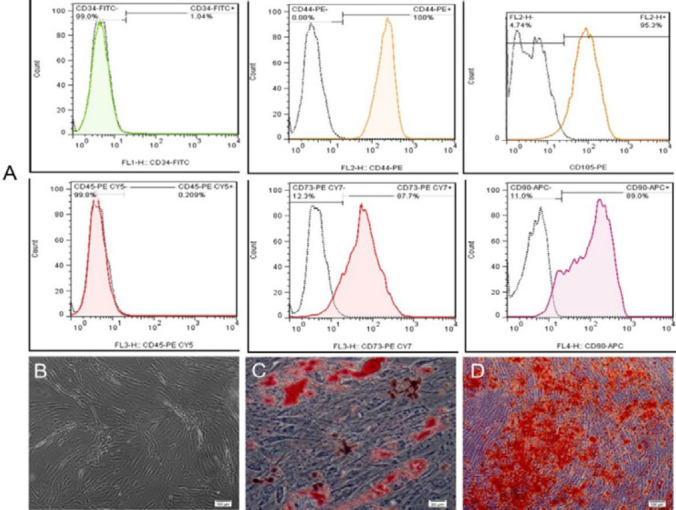
Characterization of isolated AdMSCs. A) Flow cytometric analysis of the isolated AdMSCs. The cells were positive for CD44, CD105, CD73, and CD90 surface markers while not showing surface expression of CD34 and CD45 markers. B) morphology of AdMSCs at P3 (Light microscopy). C) Oil Red staining indicative of successful adipogenic differentiation of these cells. D) alizarin red staining indicating osteogenic differentiation of AdMSCs on day 21 after osteoinduction

The isolated AdMSCs at passage 3 (P3) exhibited a spindle-shaped morphology similar to fibroblast cells indicating morphologic characteristics of undifferentiated MSCs (Figure 2B). This observation supports the successful isolation of MSCs from adipose tissue. The isolated AdMSCs were positive for CD73, CD90, CD105, and CD44 while they did not express CD34 and CD45 confirming the mesenchymal nature of these cells (Figure 2A). Also, our differentiation analyses on P3 AdMSCs were indicative of high differentiation capabilities of these cells in both osteogenic and adipogenic media (Figure 2C, D).

***MSC-EX isolation and characterization***

**Figure 3:**
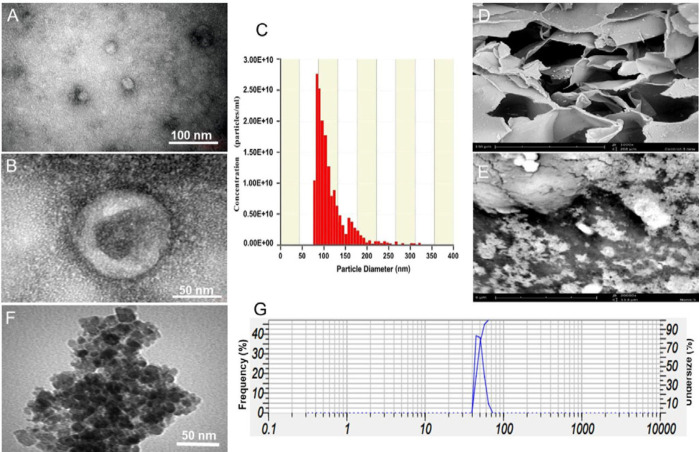
AdMSC-derived exosomes were characterized by TEM (A, B) and DLS (C). SEM images of the prepared scaffolds showing the presence of nano- and micro-sized pores under 1000X (D) and 2000X (E) magnifications. TEM and DLS analysis of CFNPs (F, G)

For exosome isolation from cultured AdMSCs, we used the Exo-spin^TM^ kit. The results of TEM analysis showed that the vast majority of the isolated particles possessed membranous cup-shaped structures similar to the previously reported morphological structure of exosomes (Figure 3A, B). Based on our DLS analysis (Figure 3C), most of the isolated nanoparticles exhibited a size range of 75–130 nm which falls within the diameter range reported for such nano-sized particles. Also, according to the Bradford assay, we could recover a mean total exosome protein content of 1168.7 µg/ml from the media.

***Scaffold characterization***

**Figure 4:**
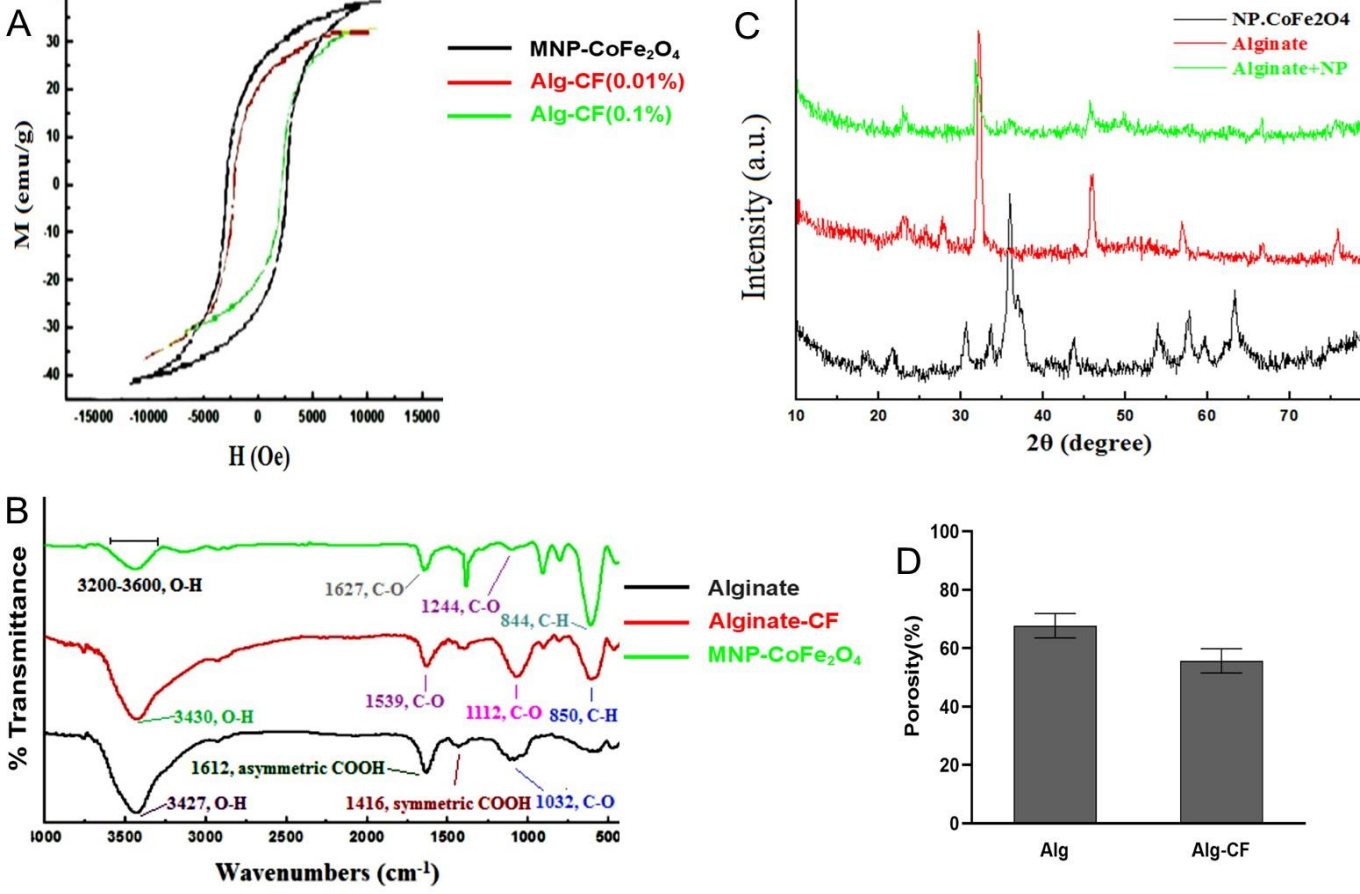
Alg and Alg-CF scaffolds were characterized by various techniques including VSM (A), FTIR (B), XRD (C), and porosity measurement (D)

To determine the structural morphology of the scaffolds, SEM images were taken from scaffold sections (Figure 3D, E). According to these images, both Alg and Alg-CF scaffolds had micro- and nano-sized pores. A variety of pores including open, closed, and interconnected pores were observed in the structure of the scaffolds. Regarding CFNPs, DLS, and TEM, analyses showed nanoparticles exhibited a size range of 44–65 nm (Figure 3F and G). The data of the VSM analyzer indicated that 0.1% CFNPs possessed acceptable magnetic properties (Figure 4A). We used FTIR spectroscopy to confirm the structure of the prepared scaffolds. The wavenumber range recorded by FTIR spectrometer scaffolds containing 0.01% (Equinox 55, Bruker, Germany) was from 400 to 4000 cm-1. To evaluate the structural impact of CFNPs on the Alg scaffold, the bare Alg scaffold and Alg-CF scaffold underwent FTIR spectroscopy. Bare Alg scaffold spectrum showed peaks around 1034, 1408, 1610, 2929, and 3427 cm^-1^ which correspond respectively to O–H bonding, symmetric and asymmetric stretching of COOH, and stretching of H–Csp3 and O–H in the polysaccharide (19, 20). FTIR of CFNPs detected peaks in the wavenumbers of 1244, 844, and 1627 cm-^1^ with a broad dip band (3000-3410 cm^-1^). This broadband was related to O–H stretching of H_2_O molecules attributed to the chemical adsorption of molecules of water at the surface of CFNPs (Figure 4B). A new peak appeared at 564 cm^− 1^, in the magnetic hydrogel that belongs to the tetrahedral group of Fe_3_^+^–O_2_^−^, and confirmed cobalt ferrite nanoparticles are scattered among hydrogels (21). Bands around 520 cm^−1^ in Fe_3_^+^–O_2_^−^ of pure cobalt ferrite nanoparticles indicate their tetrahedral and octahedral structure. XRD analysis was used to investigate crystallinity and determine phases of the prepared scaffolds. In the XRD pattern of the bare Alg scaffold, the observed broad peak in the 2θ range of 20–32° relates to the amorphous structure of sodium alginate polysaccharide. Typical halos of sodium alginate were observed at 13.7° and 21.4° which correspond respectively to 6.45 and 4.42 Å. Concerning CFNPs, the peaks were observed at 30.32°, 35.57°, 43.23°, 45.56°, 56.59° and 62.67° (Figure 4C). In this study, the porosity of Alg and Alg-CF scaffolds were calculated to be 68% and 53%, respectively (Figure 4D). the mean θ angles in Alg and Alg-CF scaffolds were estimated to be 33.33° and 13.92°, respectively. On such a basis, both of the scaffolds were shown to possess proper surfaces to interact with cells. scaffold hydrophilicity is enhanced by increases in CFNP concentration.

**Figure 5:**
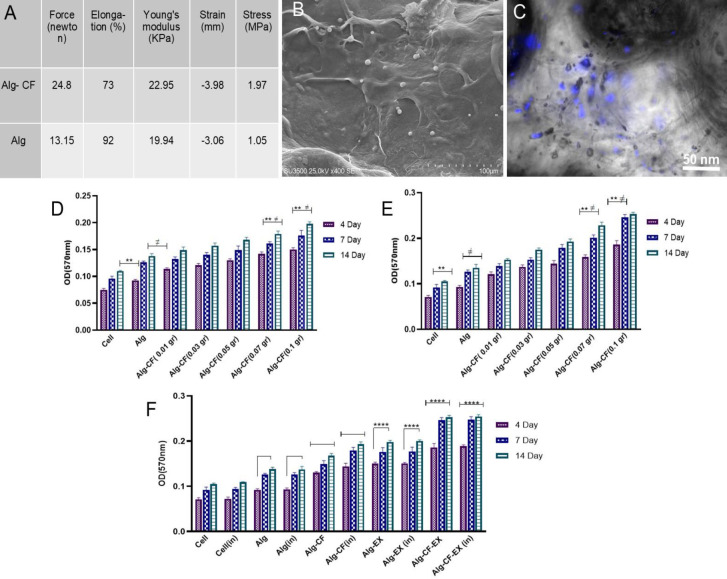
Mechanical parameters of Alg and Alg-CF scaffolds (A). SEM images showing the penetration of cultured AdMSCs in the structure of scaffolds (B). DAPI staining of cell nuclei indicating the presence of the cultured cells on scaffold surface(C). Cytocompatibility analysis of the cultured AdMSCs over time on the Alg and Alg-CF scaffolds with different concentrations of CFNPs under non-induced and induced conditions, respectively (D, E). Cytocompatibility of the cultured AdMSCs on the scaffolds after addition of MSC-EX under non-induced and induced conditions(F). *P<0.05, **P<0.01, ***P<0.001, ****P<0.0001

Compressive tests were carried out for generating a stress-strain curve and calculating Young’s modulus. Since the elastic area of the curve showed much lower stress values, the initial slope of the curve at the strain of 10% was utilized for modulus calculation. A lower Young’s modulus, which indicates a higher strain value against the exerted stress, represents a higher elastic property (Figure 5A). As demonstrated by stress-strain curves, Alg-CF scaffolds showed higher exerted force and Young’s modulus indicating that the addition of CFNPs to Alg hydrogels results in increased mechanical strength against compression. The orientation of polymer chains can be rearranged in the presence of nanoparticles leading to formation of a regular structure. Also, the mechanical features of magnetic hydrogels can be improved due to the electrostatic interaction of nanoparticles and functional groups of the polymer chain (22, 23).

***AdMSCs monitoring in the structure of scaffolds***

SEM images (Figure 5B), captured on day 7 of AdMSCs culture on Alg and Alg-CF scaffolds, showed round cells which had penetrated the scaffolds and formed a monolayer. Also, the images illustrated spindle-shaped cells covering the scaffold surface; and nuclear staining by DAPI confirmed the presence of cells growing on both scaffolds on the evaluated days (Figure 5C).

***Cell viability assay***

The viability and proliferation of AdMSCs on the synthesized scaffolds on days 4, 7, and 14 after cell seeding were evaluated by MTT assay. The results elucidated that in all groups, AdMSCs were alive at each time interval evaluated. As demonstrated in Figure 5D and E, cell proliferation increased over time and reached its maximum level on day 14. To investigate the effect of magnetic fields on AdMSC proliferation, some of the wells were stimulated by a static magnetic field (SMF). Cell viability and proliferation in the induced and non-induced groups at the mentioned time intervals did not show significant differences. This is while in both induced and non-induced groups, AdMSCs showed a higher proliferation rate when cultured on Alg-CF scaffolds compared with the cells cultured on Alg scaffold as well as the control group. Cell proliferation on Alg-CF scaffolds with CFNP concentrations of 0.7 and 0.1 gr showed statistically significant differences (*P*<0.0018) in comparison with the Alg scaffold and the control group. Based on the obtained results, an Alg-CF scaffold containing 0.1 gr CFNPs was selected for further experiments. To investigate the impact of MSC-EX on cellular proliferation on the scaffolds, MTT assays were again performed. MSC-EX was loaded on both Alg and Alg-CF (0.1 gr) scaffolds and proliferation of the cultured AdMSCs on the respective scaffolds under both non-induced and induced conditions were assessed after cell seeding. Based on Figure 5 F, we found significant differences between Alg-EX and Alg-CF-EX groups and other groups. MSC-EX induces cell proliferation on the scaffolds so the proliferation rate was shown to be higher on Alg-EX and Alg-CF-EX compared with bare Alg and Alg-CF, respectively. However, we found no statistically significant difference in AdMSC proliferation under normal conditions compared with magnetic field stimulation conditions. Though, the proliferation rate was increased under induced conditions. The results point to the remarkable pro-growth effects of MSC-EX on AdMSCs cultured on the scaffolds.

***ALP assay***

**Figure 6:**
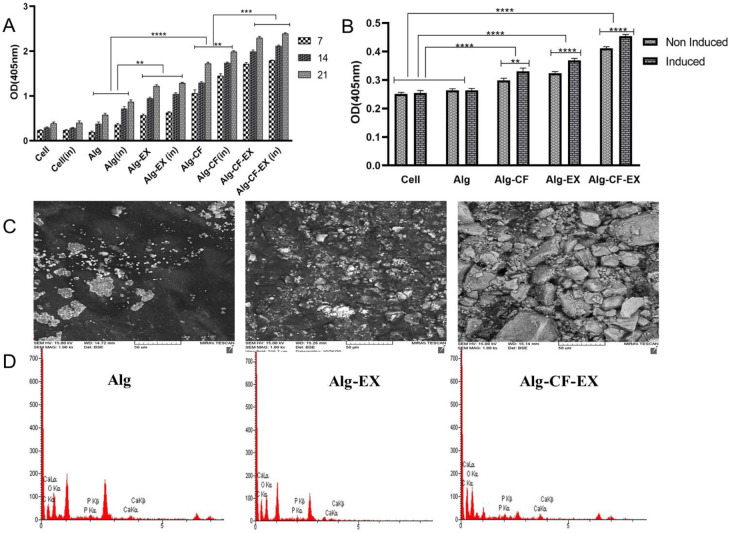
Effects of different scaffolds on the osteogenic commitment of cultured AdMSCs over time under non-induced and induced conditions as evaluated by ALP activity measurement (A) and alizarin red staining (B). SEM-EDX images confirmed the presence of calcium deposits in the Alg-CF-EX scaffold and higher matrix mineralization(C). The energy dispersive X-ray (EDX) analysis of groups(D). Alg alginate; Alg-EX: alginate- Exosomes; Alg-CF-EX: Alginate -Cobalt ferrite- Exosomes. **P<0.01, ****P<0.0001

ALP activity, as one of the early markers of osteogenic differentiation [20], was measured 7, 14, and 21 days after addition of osteogenic media. At all time intervals evaluated, ALP activity was significantly higher in Alg-CF, Alg-EX, and Alg-CF-EX wells compared with Alg and negative control wells (*P*<0.0001). In both types of scaffolds, that is Alg and Alg-CF, ALP activity increased in a time-dependent manner. As shown in Figure 5D, magnetic field stimulation of Alg-CF and Alg-CF-EX scaffolds caused significantly higher expression of ALP on days 14 and 21 as compared with the corresponding non-stimulated scaffolds (*P*<0.0001). Also, MSC-EX loading promoted ALP activity. Comparison of ALP activity in the scaffolds with and without EX showed that at all time intervals, MSC-EX loading significantly showed increased ALP activity (*P*<0.0001). These results show synergistic effects of CFNPs and MSC-EX on the enhancement of ALP activity and hence the osteogenesis process so that the scaffolds containing both CFNPs and EX (Alg-CF-EX) exhibited the highest level of ALP activity under induced conditions (Figure 6A). 

***Alizarin red staining***

This analysis was conducted on day 21 post osteoinduction to stain calcium deposits produced by differentiated cells. Both Alg and Alg-CF scaffolds under non-induced and induced conditions showed calcium deposition. This indicates that the cultured cells had successfully differentiated into osteoblast-like cells. mineralization was significantly higher in Alg-CF, Alg-EX, and Alg-CF-EX wells compared with Alg and negative control wells (*P*<0.0001). Also, EX loading was found to enhance matrix mineralization, so that calcium deposition reached its maximum level in the Alg-CF-EX group. According to Figure 6 B, calcium deposition was higher in induced compared with non-induced scaffolds in Alg-CF (*P*<0.0015), Alg-EX, and Alg-CF-EX (*P*<0.0001) wells. In the alizarin red staining analyses, we could apprehend the mentioned synergy in the Alg-CF-EX group. 

***SEM-EDX analysis***

EDX analysis (with more focus on P and Ca elements which are involved in the formation of mineral deposits during osteogenesis), confirmed the presence of higher calcium deposits in Alg-CF compared with Alg scaffolds) Figure 6C, D (. Also, magnetic stimulation caused more calcium deposition in stimulated scaffolds in comparison with the corresponding non-stimulated scaffolds (data not shown here). Again, calcium and phosphorus deposits were highest in the Alg-CF-EX group. 

## Discussion

In the current study, we assessed the potential of an alginate-based bone engineering scaffold containing CFNPs and MSC-EX. To the best of our knowledge, this is the first study to examine the osteogenic differentiation ability of a synthesized tissue-engineered scaffold incorporating three different components of alginate hydrogel, CFNPs, and MSC-EX. Cell proliferation was found to be higher on Alg-EX and Alg-CF-EX when compared with Alg and Alg-CF, respectively, bringing to mind the notion that AdMSC-EX might be a more potent inducer of cell proliferation than CFNP. Then, we investigated the Osteogenic differentiation properties of the scaffolds by ALP assay, alizarin red staining, and SEM-EDX analysis. Based on our results the highest activity of ALP was observed in the Alg-CF-EX scaffold indicating the positive synergistic effects of CFNPs and MSC-EX on the ALP activity and hence the osteogenesis process. The alizarin red test comparison between the potential of MSC-EX and CFNPs suggests that MSC-EX induces a higher calcium deposition as we found a higher calcium deposition in the Alg-EX group compared with Alg-CFNP group even in the presence of the magnetic field. Finally, the results of SEM-EDX analysis showing higher levels of calcium and phosphorus in the Alg-CF-EX group were in line with the alizarin red test and corroborated the highest level of osteogenic induction when the three different elements of hydrogel, MNP, and exosome are combined (Figure 6).

In the field of regenerative medicine, we require a synthetic extracellular matrix to support stem cells during their differentiation and integration into surrounding tissue(24). In this regard, alginate hydrogel possesses material properties that allow manipulation in various ways for several applications such as 3D printing, drug/growth factor delivery, injectable fillers, cell encapsulation, and so forth(25). In the current study, we also used alginate hydrogel as a cell-supporting scaffold for *in vitro* osteogenic differentiation of AdMSCs. Another arm of our synthesized scaffold is related to MNP and magnetic field. Previous studies have focused mainly on iron oxide MNPs. These MNPs have some limitations including weak magnetic properties under physiological conditions and at small sizes. In the present study, we utilized CFNPs. In comparison with iron oxide MNPs, CFNPs have excellent mechanical hardness and physiochemical characteristics with higher stability and colloidal dispersibility at physiological conditions(16). In 2019. Filippi *et al*. developed magnetic nanocomposite hydrogels and these magnetic hydrogels were assessed for enhancing ALP activity, increasing osteogenesis genes expression, and improving the formation of mineralized extracellular matrix (ECM) under both *in vitro* and *in vivo* conditions. Their findings indicated that SMF-stimulated cell-laden hydrogels exhibited higher mineralization as well as faster vascularization (which is needed for bone reconstruction) compared with MNPs or SMF alone(26). In another study, they designed a magnetic composite scaffold comprising polycaprolactone (PCL), mesoporous bioactive glass (MBG), and magnetite (Fe_3_O_4_) by a three-dimensional (3D) bioplotter. It was shown that this nanocomposite scaffold significantly induced the proliferation of human bone marrow-derived stem cells (BMSCs), ALP activity, ECM mineralization, and expression of osteogenic genes (27). MNPs, mostly superparamagnetic iron oxide nanoparticles (SPIONs), alone or in combination with a magnetic field have been shown to improve osteogenic differentiation of stem cells and augment *in vivo* bone regeneration (28, 29). Iron oxide nanoparticles have been shown to promote osteogenic differentiation of BMSCs through induction of the mitogen-activated protein kinase (MAPK) pathway (15). In a study by Yun and colleagues, PCL-MNP nanocomposite scaffolds were investigated. The researchers could manifest the synergy between the magnetic scaffold and magnetic force via initiation of integrin signaling pathways involving focal adhesion kinase, RhoA, paxillin, MAPK, and nuclear factor kappa B (NF-κB) and promotion of bone morphogenetic protein-2 (BMP-2) as well as Smad1/5/8 phosphorylation. In the model of mouse calvarial defect, application of SMF conspicuously improved new bone formation at 6 weeks. Combined application of 10% MNP and SMF increased bone volume. This observation was ascribed to the synergy of BMP, integrin, NF-κB, and MAPK signaling pathways in the osteoblasts when scaffolds were combined with SMF. In this line, the results of our ALP assay could shed light on the existence of a similar synergy between SMF and CFNPs (Figure 6A). Although, in the alizarin red staining analyses, we could apprehend the mentioned synergy only in the Alg-CF-EX group (Figure 6B). The magnitude of the SMF that we applied in our study was 125 mT which falls within the range of moderate intensity SMF (30). In previous studies, SMF exposure has been shown to increase significantly the MSCs proliferation rate, ALP activity, matrix mineralization, as well as the expression of osteogenic proteins such as osteocalcin (OCN) and osteopontin (OPN), suggesting that moderate-intensity SMF can enhance MSC proliferation and osteoblastic differentiation. However, according to MTT assays in our study, we did not observe significant effects of SMF on AdMSCs proliferation on the prepared scaffolds (Figure 5D and 5E). In agreement with this result as to the effect of magnetic field on cellular proliferation, in a study exploring proliferation rates of BMSCs cultured on a magnetic scaffold composed of Fe_2_O_3_, nano-hydroxyapatite (n-HA), and polylactic acid (PLA), it was demonstrated that addition of MNPs and the use of a pulsed electromagnetic field (PEMF) did not affect the proliferation rate while MNPs combined with PEMF exerted a positive effect on osteogenic differentiation of BMSCs (31). Some possible mechanisms through which SMF exerts the mentioned effects have been suggested. Cellular membranes possess diamagnetic properties and SMF exposure is assumed to modify the cell membrane flux. Furthermore, ECM proteins have diamagnetic properties as well and SMF is suggested to affect their structures and orientations (32, 33). Also, in the study of Petecchia and coworkers, it was shown that PEMF promotes osteogenic differentiation of MSCs by selectively acting on Ca^2+^-related mechanisms (34). The last arm of our scaffold was MSC-EX. We figured out that MSC-EX loading of scaffolds significantly enhanced both cell proliferation and markers of osteogenic differentiation including ALP activity and calcium deposition. Exosomes can be contemplated as important paracrine mediators capable of being used as therapeutic tools for tissue repair, particularly in the bone regeneration field (35, 36). Exosomes can function within the target area because of their specific features including biocompatibility, stability, low toxicity, permeability, and low immunogenicity (37). Gandolfi *et al*. in 2020, devised scaffolds made up of PLA and various percentages of bioactive materials enriched with human AdMSC-derived exosomes. Based on the findings of this research group, exosomes could interact with AdMSCs through membrane-membrane fusion thereby delivering their molecular contents into these cells and triggering osteogenic differentiation. Incubation of AdMSCs in the presence of exosome-enriched scaffolds resulted in increased expression of osteonectin, OCN, type 1 collagen, and Runx which are all biochemical markers of osteogenesis (18). In another study by Zhang and colleagues, exosome/ tricalcium phosphate(β-TCP) combination scaffolds were found to significantly enhance osteogenesis relative to pure β-TCP scaffolds. Based on their *in vitro* studies, exosomes could be released from β-TCP and internalized by BMSCs. This internalization was shown to significantly augment the proliferation, migration, and osteogenic commitment of these cells. Their mechanistic evaluation unveiled the critical involvement of the PI3K/Akt signaling pathway in mediating the mentioned osteoinductive activity of the exosome/ β-TCP scaffold(6). In line with this study, it was revealed by Baker *et al*. that MSC osteoinductive function is mediated through PI3K/Akt pathway activation (38). Recently, the power of human bone marrow mesenchymal stem cell-derived exosomes (BMMSC-EX) in healing bone fracture was studied by Zhang *et al*. in a rat model of nonunion. In their study, BMMSC-EX transplantation clearly promoted osteogenesis, angiogenesis, and the process of bone healing. It was found that BMMSC-EX accelerated the proliferation and migration of osteoblast and endothelial cells. These exosomes were shown to activate BMP-2/Smad1/RUNX1 and HIF-1α-VEGF signaling pathways in osteoblasts and endothelial cells, respectively, to promote osteogenesis and angiogenesis for enhanced fracture healing (39).

Alg: Alginate; Alg-CF: Alg-cobalt ferrite; VSM: Vibrating sample magnetometer; FTIR: Fourier transform infrared spectroscopy; XRD: X-ray diffraction

Alg: Alginate; MSC-EX: Mesenchymal stem cell-derived exosomes; Alg-CF: Alginate-cobalt ferrite; Alg-EX: Alginate- Exosomes; Alg-CF-EX: Alginate -cobalt ferrite- exosomes

Alg: Alginate; MSC-EX: Mesenchymal stem cell-derived exosomes; Alg-CF: Alginate -cobalt ferrite; Alg-EX: Alginate-Exosomes; Alg-CF-EX: Alginate -Cobalt ferrite- Exosomes

## Conclusion

Collectively, MNPs-incorporated alginate scaffolds were synthesized and characterized by various techniques. It was found that Alg and Alg-CF (with CFNP concentration range of 0.01 to 0.1 gr) scaffolds were not only cytotoxic but also supported the viability/proliferation of the cultured AdMSCs. We provided evidence that magnetic composites containing exosomes can be more effective. In the current study, we developed a novel cell-free bone engineering scaffold composed of three distinct components of Alg hydrogel, CFNPs, and MSC-EX and evaluated *in vitro* potential of its osteoinduction. We showed that the combined synergy of these elements results in a more prominent osteogenic response; alkaline phosphatase activity and mineralization were clearly seen as well. under SMF stimulation. Our study represents a new horizon in the construction of bioengineered scaffolds for bone regeneration purposes. 

## Authours’ Contributions

FS Contributed to all experimental work, drafting, and statistical analysis. SH and MS Provided scientific consulting and contributed to concept and design. AJ Contributed to the experimental work and data collection. HN Provided scientific consulting, English and scientific review and editing, and final approval of the manuscript. AKH was responsible for overall supervision. All authors read and approved the final manuscript.

## Conflicts of Interest

The authors have no conflicts of interest.
